# Surgical management for catheter-induced pulmonary artery injury during cardiac surgery: a case report

**DOI:** 10.1186/s44215-025-00227-0

**Published:** 2025-10-21

**Authors:** Harunobu Sasanuma, Masaaki Nagano, Mamoru Muto, Tatsuo Maeyashiki, Nobuyuki Inoue, Satoshi Nagasaka

**Affiliations:** 1 Department of Thoracic Surgery, Center Hospital of the National Center for Global Health and Medicine, 1-21-1 Toyama, Shinjuku-ku, Tokyo 162-8655 Japan; 2 Department of Cardiovascular Surgery, Center Hospital of the National Center for Global Health and Medicine, 1-21-1 Toyama, Shinjuku-ku, Tokyo 162-8655 Japan

**Keywords:** Cardiac surgery, Pulmonary artery catheter, Pulmonary artery injury, Surgical treatment

## Abstract

**Background:**

A pulmonary artery catheter (PAC) is widely used to manage various health conditions during cardiac surgery. However, it can cause rare but life-threatening complications such as pulmonary artery injury. Although surgical treatment is often required for pulmonary artery injury caused by a PAC, detailed reports of the surgical management of this complication are lacking. We present a case of PAC-induced pulmonary artery injury that occurred during cardiac surgery and was successfully managed with surgical treatment without pulmonary resection.

**Case presentation:**

A 76-year-old woman with severe heart valve disease underwent replacement of the aortic and mitral valves and surgical repair of the tricuspid valve using a PAC. Massive bleeding into the airway occurred during withdrawal from cardiopulmonary bypass. Fluoroscopic contrast injection through the PAC identified a PAC-induced tear in the left pulmonary artery as the cause of the bleeding. Opening of the left fourth intercostal space was performed without changing the supine position of the patient. Bleeding was successfully controlled by ligating the damaged branch of the pulmonary artery (A6c) and suturing the roots of the peripheral pulmonary arteries (A9 and A10) without performing lung resection. The patient recovered without complications and was discharged in good condition.

**Conclusions:**

Although PAC-induced pulmonary artery injury is associated with a high mortality rate, the patient survived cardiac surgery with lung preservation in the supine position.

## Background

A pulmonary artery catheter (PAC) is an important device that can be used to intraoperatively and perioperatively monitor cardiac surgery and manage critically ill patients [1]. Although the PAC provides significant clinical benefits, it is associated with complications such as infection, pneumothorax, arrhythmia, cardiac perforation, vena cava injury, right heart cavity injury, and pulmonary artery injury [2]. In particular, pulmonary artery injury is a rare complication with a frequency of 0.01% to 0.47% and a mortality rate of 50% to 70% [3].

Although mechanical ventilation and coil embolization may be used to treat pulmonary artery injury [4], surgical treatment should be actively considered if unstable hemodynamics or central arterial bleeding is suspected. In some cases, the lungs can be preserved using pulmonary artery sutures and ligation; however, pneumonectomy or lobectomy is often required for extensive intralobar bleeding or intrathoracic bleeding [5].

Detailed reports of the surgical management of PAC-induced pulmonary artery injury are scarce. Therefore, we present a case of PAC-induced pulmonary artery injury that occurred during cardiac surgery and was successfully managed with surgical treatment without pulmonary resection.

## Case presentation

A 76-year-old woman with worsening dyspnea and leg edema was referred to our cardiology department. A thorough examination revealed united valvular disease (aortic, mitral, and tricuspid regurgitation), persistent atrial fibrillation, and chronic heart failure. After heart failure management, aortic valve replacement, mitral valve replacement, and tricuspid annuloplasty were planned.

**Fig. 1:**
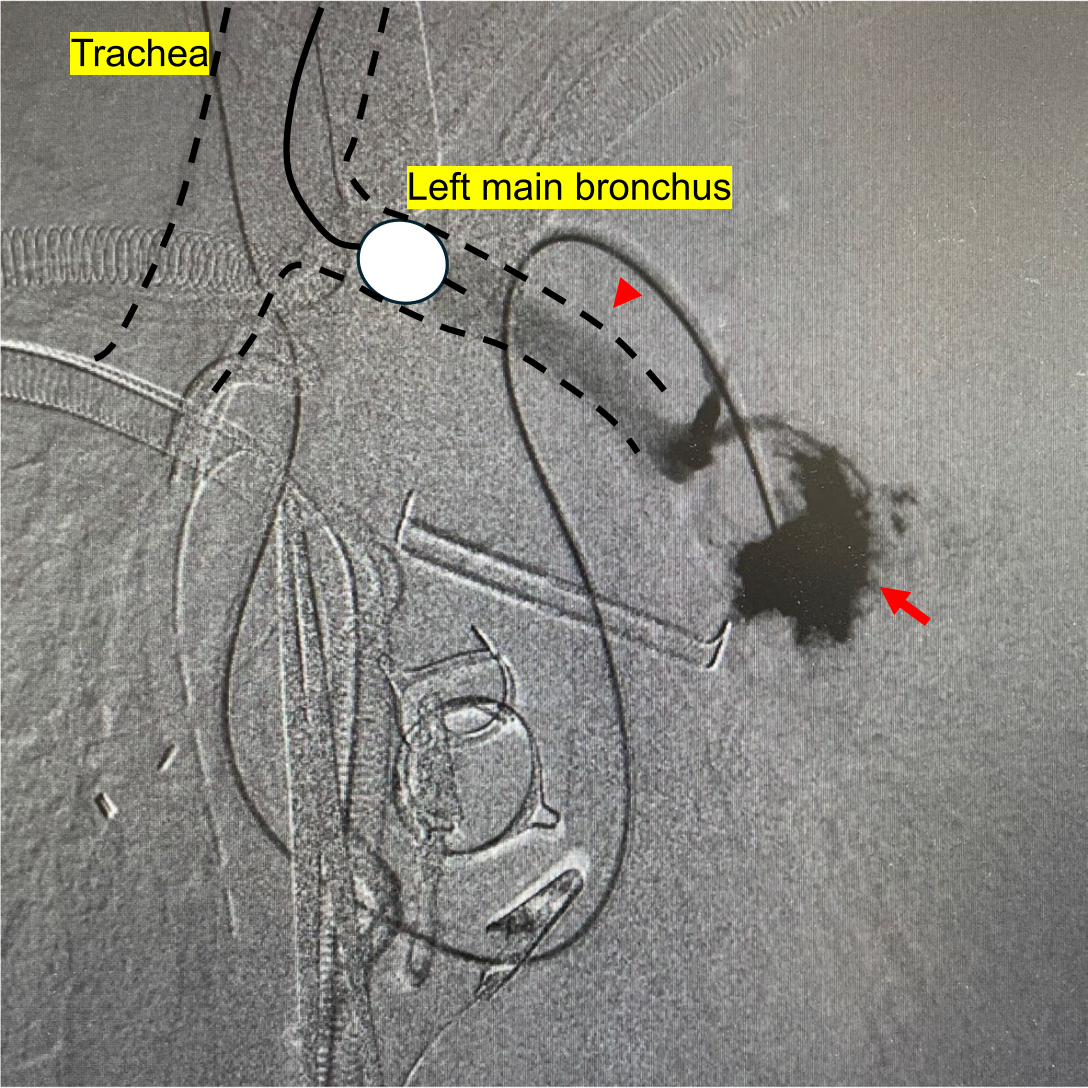
Pulmonary angiography from the pulmonary artery catheter showed the massive extravasation of contrast medium from the left pulmonary artery (arrow) and flow into the left main bronchus via the bronchioles (arrowhead). A bronchial blocker was placed in the left main bronchus

**Fig. 2:**
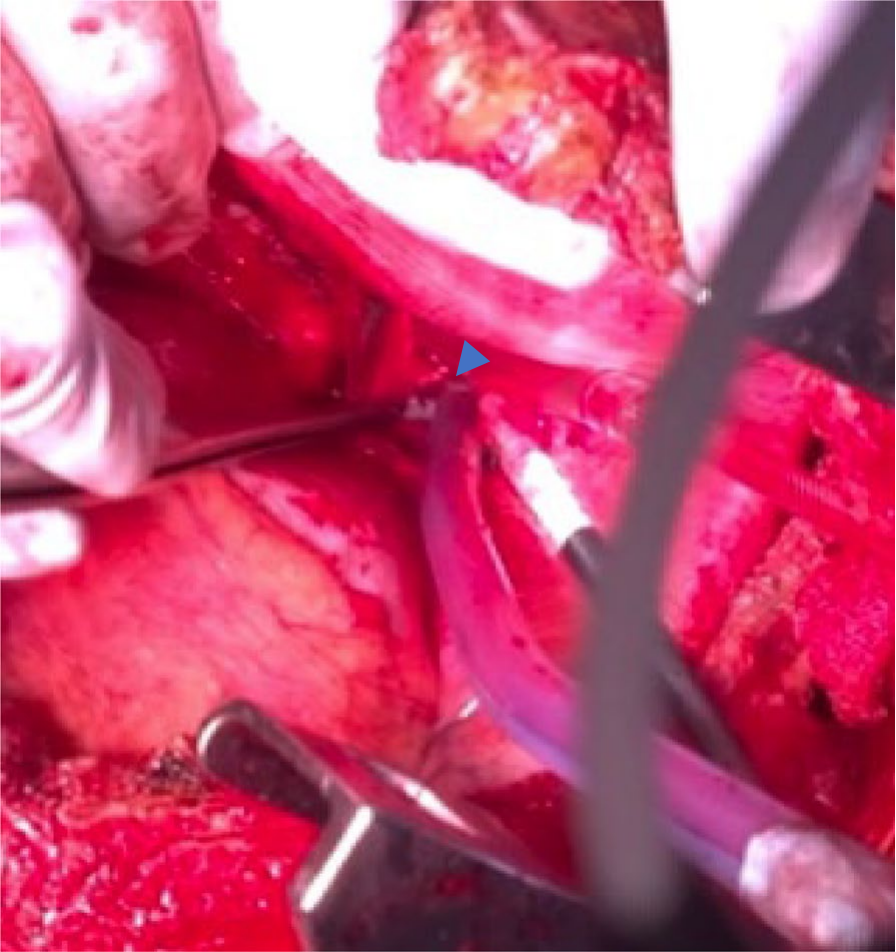
Intraoperative findings revealed a large tear of A6c (arrowhead)

**Fig. 3:**
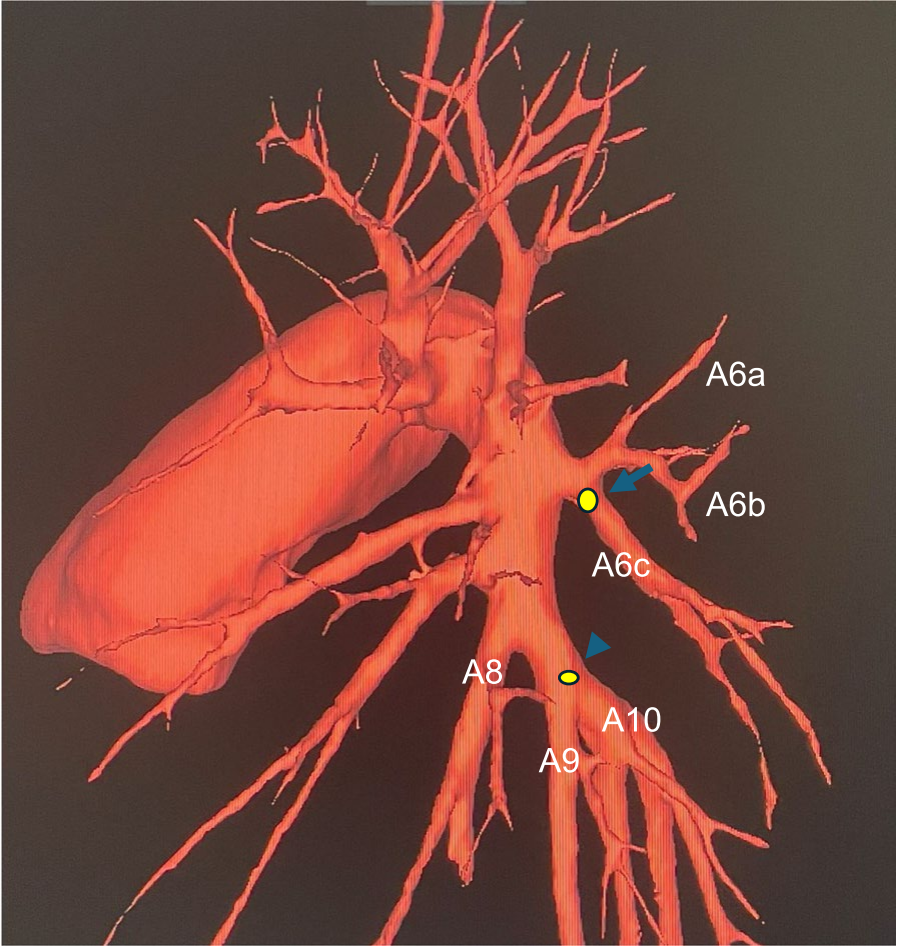
A large tear of the lower lobe of the superior segmental pulmonary artery (A6c) (arrowhead), and a small defect at the roots of the basal segmental pulmonary arteries (A9 and A10) (arrow)

After anesthesia induction, the anesthesiologist inserted a PAC. At our hospital, we usually confirm the position of the PAC tip using both the pressure waveform and transesophageal echocardiography. However, in this case, we had to place the PAC using only the pressure waveform because of the poor echocardiography findings. Following a median sternotomy, cardiopulmonary bypass (CPB) was instituted with arterial perfusion via the ascending aorta and venous drainage through the superior and inferior vena cava. The procedure was then performed under total bypass, including mitral valve replacement, aortic valve replacement, and tricuspid annuloplasty. A balloon was inflated and guided toward the pulmonary artery because the PAC was bent and protruded from the right atrium. When the right atrium was closed and weaning from CPB was initiated, a large amount of blood gushed from the tracheal tube. Immediately, we stopped weaning and opened the bilateral chest through the midline incision. Hemorrhage was observed in the left chest cavity and left lower lobe of the lung. A left bronchial blocker was promptly inserted. We suspected a PAC-induced pulmonary artery injury and performed fluoroscopic contrast injection through the PAC using a portable C-arm, confirming extravasation and contrast leakage into the left main bronchus via the bronchioles (Fig. 1). A pulmonary artery injury and pulmonary artery–bronchial fistula were diagnosed, and opening of the left fourth intercostal space was performed via a median incision while the patient remained in the supine position. The left main pulmonary artery trunk was secured by opening the pericardial sac, and a large tear of the lower lobe of the superior segmental pulmonary artery was confirmed as the damaged area (A6c) (Figs. 2, 3). Because of difficulty repairing the injury, the central side was closed with continuous sutures using 5-0 monofilament, and the peripheral side was ligated with 2-0 silk sutures to stop the bleeding. A small defect was observed at the roots of the basal segmental pulmonary arteries (A9 and A10) and directly repaired using 5-0 monofilament (Fig. 3). Because the hematoma in the lower lobe was small and improved after hemostasis, we considered that the lower lobe could be preserved; therefore, treatment of the injured area was completed using fibrin glue and a polyglycolic acid sheet. Thereafter, the patient was successfully weaned from CPB, and surgery was completed. The operative time was 9 h and 47 min, and the blood loss volume was 2032 mL. The repair was completed within 1 h and 9 min of diagnosing the injury.

The patient was extubated on postoperative day 6. The thoracic drain was removed on postoperative day 15. On postoperative day 53, the patient was discharged. A follow-up examination including chest computed tomography showed no evidence of pulmonary necrosis or infection.

## Discussion and conclusions

We encountered a case of a PAC-induced left pulmonary artery injury that was diagnosed based on bleeding in the airway after the patient was weaned from CPB. Treatment comprising right single-lung ventilation was quickly performed while the patient remained in the supine position.

We obtained a good field of view by opening the left fourth intercostal space while the patient was in the supine position and experienced ease when approaching the bleeding site. A PubMed search using the terms “pulmonary artery catheter,” “pulmonary artery rupture,” and “surgical treatment” identified six reports of surgical treatment for PAC-induced pulmonary artery injury (Table 1). Of these cases, two involved treatment in the lateral decubitus position [6, 7] and one involved treatment in the supine position [7] . While the injuries were identified postoperatively in the two patients who underwent treatment in the lateral decubitus position, the injury was identified intraoperatively in the patient who underwent treatment in the supine position, similar to our case. Although the lateral decubitus position provides a familiar field for thoracic surgeons, transitioning from the supine position to the lateral decubitus position during cardiac surgery is time-consuming and potentially detrimental in urgent scenarios. In contrast, the supine position facilitates immediate thoracic access during cardiac surgery. Additionally, CPB allows the patient to maintain respiratory circulation, thus providing time for surgical treatment of the injured site and reducing blood loss by reducing the amount of circulating blood in the lungs [5]. Therefore, performing surgical procedures with patients in the supine position may be beneficial. If a PAC-induced pulmonary artery injury occurs during cardiac surgery, as in the present case, then opening the intercostal space while the patient is in the supine position may be useful.

**Table 1:** Reports of pulmonary artery injury caused by the pulmonary artery catheter during or after surgery

Author	Year	Situation	Procedure	Bleeding point	Surgical treatment	Position	Outcome
Deren [1]	1979	Intraoperative	AVR	Unknown	Right PA repair	Unknown	Dead
Muller [8]	1985	Postoperative	MVP and CABG	Unknown	Right PA repair	Unknown	Alive
Reber [9]	1996	Intraoperative	CABG	Right A4	Right middle lobectomy	Unknown	Alive
Sekkal [7]	1996	Intraoperative	AVR and MVR	Unknown	Right middle lobectomy	Supine position	Alive
Sekkal [7]	1996	Postoperative	AVR and CABG	Unknown	Right middle lobectomy	Left lateral decubitus position	Alive
Booth [6]	2012	Postoperative	CABG	Truncus superior A	Right PA repair	Left lateral decubitus position	Alive
Our report	2025	Intraoperative	AVR, MVR and TAP	Left a6c	Left A6c ligation	Supine position	Alive

In this case, the injury occurred in the peripheral pulmonary artery, thus enabling successful hemostasis through arterial repair and ligation without lung resection. Table 1 describes pulmonary artery repair performed for three previous cases [1, 6, 8] and lobectomy performed for three other previous cases [7, 9]. In the three cases with a PAC-induced pulmonary artery injury during cardiac surgery [1, 7, 9], only one case was treated with pulmonary artery repair alone [1]. We decided to preserve the left lung of our patient by resecting A6c because collateral blood circulation from the bronchial and pulmonary arteries can prevent pulmonary necrosis. No color change in S6 occurred after A6c ligation was performed for our patient. Achieving hemostasis using suture repair alone in the central pulmonary artery (main trunk or lobar level) is often difficult, and lung resection may be necessary [10]. However, in peripheral branches (segmental or subsegmental level), hemostasis may be achievable with pulmonary artery repair alone [5]. When low functioning is possible because of extensive intrapulmonary bleeding, or when there is a high risk of lung necrosis, careful consideration is required because lung preservation may be counterproductive. However, preserving the lungs by treating only the damaged blood vessels should be considered if possible.

Establishing one-lung ventilation using a bronchial blocker greatly affects the stability of breathing and circulation. Four previous cases of PAC-induced pulmonary artery injury during cardiac surgery, including our case, exhibited tracheal bleeding at the time of CPB weaning (Table 1), which was likely caused by the increased pulmonary blood flow during the transition from total to partial extracorporeal circulation. Additionally, patients receive systemic heparin during CPB, thus raising concerns about massive tracheal bleeding. Therefore, it is crucial to quickly identify the bleeding bronchus and prevent blood from entering the healthy bronchus by promptly applying a bronchial blocker [11]. Bilateral thoracotomy of our case confirmed that the bleeding originated from the left lung. Therefore, a bronchial blocker was immediately applied in the left main bronchus, resulting in a minimal effect on the right lung and early postoperative extubation. When pulmonary artery injury is suspected, the rapid application of a bronchial blocker to protect the healthy lung can help stabilize the respiratory status and contribute to early recovery.

Although a PAC-induced pulmonary artery injury was suspected, identification of the injury site was difficult because of insufficient visibility from the median incision. Additionally, observation of the injury site using a bronchoscope was difficult because of excessive bleeding in the trachea. Therefore, pulmonary artery angiography was performed using a portable C-arm to confirm the exact bleeding point location and determine the best hemostasis method (i.e., whether to change the patient’s position or perform additional thoracotomy). Portable C-arms are available at most facilities and are useful even when there is no time for computed tomography or catheter examinations.

In conclusion, although PAC-induced pulmonary artery injury is rare, it is life-threatening and requires an immediate diagnosis and treatment. This case provides valuable insights regarding effective treatment strategies for a PAC-induced pulmonary artery injury.

## Data Availability

The datasets supporting the conclusions of this article are included within the article.

## Abbreviations

PAC: Pulmonary artery catheter
CPB: Cardiopulmonary bypass
AVR: Aortic valve replacement
MVP: Mitral valve plasty
CABG: Coronary artery bypass grafting
MVR: Mitral valve replacement
TAP: Tricuspid annuloplasty
PA: Pulmonary artery
